# The cardiovascular phenotype of adult patients with phenylketonuria

**DOI:** 10.1186/s13023-019-1188-0

**Published:** 2019-09-06

**Authors:** Aline Azabdaftari, Markus van der Giet, Mirjam Schuchardt, Julia B. Hennermann, Ursula Plöckinger, Uwe Querfeld

**Affiliations:** 1 Department of Pediatrics, Division of Gastroenterology, Nephrology and Metabolic Diseases, Charité – Universitätsmedizin Berlin, Campus Virchow-Klinikum, Augstenburger Platz 1, 13353 Berlin, Germany; 2 Department of Nephrology, Charité – Universitätsmedizin Berlin, Campus Benjamin Franklin, Hindenburgdamm 30, 12203 Berlin, Germany; 3 Villa Metabolica, Department of Pediatric and Adolescent Medicine, University Medical Center Mainz, Langenbeckstr, 1, 55131 Mainz, Germany; 4 Interdisciplinary Center of Metabolism: Endocrinology, Diabetes and Metabolism, Charité – Universitätsmedizin Berlin, Campus Virchow-Klinikum, Augustenburger Platz 1, 13353 Berlin, Germany

**Keywords:** Phenylketonuria, Cardiovascular risk factors, Endothelial dysfunction, Oxidative stress, Vascular stiffness

## Abstract

**Background:**

Patients with Phenylketonuria (PKU) are exposed to multiple cardiovascular risk factors, but the clinical significance of these abnormalities is yet unknown. The purpose of this study was to characterize the cardiovascular phenotype in adult patients with PKU by clinical and dietary data, measurements of biochemical markers, and non-invasive examination of vascular functions.

**Results:**

Twenty-three adult patients with PKU (age: 18–47 y; 30.8 ± 8.4 y) and 28 healthy controls (age: 18–47 y; 30.1 ± 9.1 y) were included in this study. PKU patients had significantly higher systolic and diastolic blood pressure, increased resting heart rate and a higher body mass index. Total cholesterol and non-HDL cholesterol levels were significantly increased in PKU patients, whereas plasma levels of HDL cholesterol and its subfraction HDL2 (but not HDL3) were significantly decreased. The inflammatory markers C-reactive protein and serum amyloid A protein and the serum oxidative stress marker malondialdehyde were significantly higher in patients with PKU. Venous occlusion plethysmography showed marked reduction in post-ischemic blood flow and the carotid to femoral pulse wave velocity was significantly increased demonstrating endothelial dysfunction and increased vascular stiffness.

**Conclusions:**

This study shows that the cardiovascular phenotype of adult PKU patients is characterized by an accumulation of traditional cardiovascular risk factors, high levels of inflammatory and oxidative stress markers, endothelial dysfunction and vascular stiffness. These data indicate the need for early cardiovascular risk reduction in patients with PKU.

## Background

Phenylketonuria (PKU) is an autosomal-recessive inborn error of phenylalanine (Phe) metabolism, caused by the deficiency of the enzyme phenylalanine hydroxylase (PAH). In untreated patients, the accumulation of Phe and its metabolites leads to mental retardation and other neurological symptoms [1]. The objective of PKU-treatment is to lower Phe blood concentrations in order to prevent these symptoms. Thus, early diagnosis, usually via newborn screening, and immediate initiation of a strict, life-long low-phenylalanine diet is recommended [1–3]. This diet consists of a low natural protein intake supplemented with a synthetic Phe-free amino acid formula [1].

Previous studies have shown a high prevalence of cardiovascular risk factors in young patients with PKU. Among the traditional risk factors, dyslipidemia, obesity, and hypertension are the most prevalent [4–7]. Moreover, oxidative stress, hyperhomocysteinemia and proteinuria [5, 6, 8] have been reported in PKU patients and may further increase their susceptibility for cardiovascular complications [8–10]. Adult patients with low adherence to the Phe-free diet and therefore high Phe plasma concentrations seem to have higher levels of various cardiovascular risk factors [7, 8, 11]. Thus, PKU patients are exposed to multiple cardiovascular risk factors, but their impact on vascular disease is currently unknown.

The purpose of this study was to further characterize the cardiovascular phenotype in adult patients with PKU by clinical and dietary data, measurements of biochemical markers, and non-invasive examination of vascular functions. Venous occlusion plethysmography (VOP) was used to evaluate endothelial function, and the carotid to femoral pulse wave velocity (PWV) to assess the arterial stiffness.

We hypothesized that these measurements might detect distinct vascular alterations already in early adulthood and, together with clinical data and selected biochemical markers, improve the cardiovascular risk assessment of PKU patients.

## Methods

### Study population

Early diagnosed patients with PKU aged 18 years and older who were on a lifelong Phe-restricted diet with synthetic amino acid supplementation were eligible for the study. Exclusion criteria were inability to cooperate with study requirements, acute infections, smoking and pregnancy. All examinations were performed on the same day and consisted of a standardized interview, measurements of weight, height, resting heart rate, blood pressure, blood drawing (after an overnight fast), measurement of vascular stiffness by PWV and endothelial function by VOP.

The patient’s daily natural protein intake was estimated by review of the regularly conducted three-day-dietary-protocols. Total daily protein intake was defined as the sum of synthetic protein and natural protein intake.

### Biochemical analyses

All analyses were performed on venous blood samples taken under fasting conditions. Phe and tyrosine concentrations in plasma were determined by amino acid analyzer. Renal function was assessed by the estimated glomerular filtration rate (eGFR) based on the CKD-EPI equation [12].

For all other parameters, serum aliquots were frozen at − 80 °C until analysis. Serum concentrations of total cholesterol, HDL cholesterol (HDLc) and LDL cholesterol (LDLc) were measured using commercially available assay kits (Diasys, Holzheim, Germany), a microtiter plate and a spectral photometer device (Skan IT software, version 2.4.4, Thermo Scientific, Langenselbold, Germany). To obtain the HDL2c/3c ratio, the HDL3c fraction of the serum was precipitated using the Quantolip reagent B (Technoclone, Wien, Austria) and HDL2c was calculated. Serum amyloid A (SAA), C-reactive protein (CRP), apolipoprotein A1 (apoA1) and myeloperoxidase (MPO) were determined using the Luminex™ technique with commercially available assay kits (Merck Millipore, Darmstadt, Germany) and measurements were performed on a Bio-Plex® device with Bio-Plex® software (version 6.1) (Biorad, München, Germany). Malondialdehyde (MDA) and 3-nitrotyrosine (3-NT) were determined in serum by colorimetric-based enzyme-linked immunosorbent assay (ELISA) according to the manufacturer’s protocol (Cloud-Clone Corp., Houston, TX, USA).

The urine protein-to-creatinine ratio was measured in all patients by immunoturbidimetry.

### Vascular studies

Blood pressure and resting heart rate were measured with a Dinamap® Pro 100 V2 patient monitor after 10 min of rest in supine position. The mean values of two measurements were used.

Venous occlusion plethysmography was performed with the Compactus device (Sogut Medical, Königsdorf, Germany) as previously described [13]. Patients were supine for at least 10 min prior to the examination, ankles and legs were positioned on foam blocks above heart level. Blood flow measurements were performed on both lower legs using a strain gauge, detecting volume differences, while pneumatic occlusion cuffs connected to the Compactus device temporarily interrupted the blood flow on the upper legs. Measurement of the leg baseline blood flow (LBF in ml blood/100 ml leg volume /min) was performed by inflating the cuffs to 60 mmHg for 5 s and thereby occluding the venous blood flow. The average of 6–9 recordings was used for the analysis. To measure the post-ischemic blood flow, 3 min of ischemia were induced by inflating the cuffs to 180 mmHg or 50 mmHg above the systolic blood pressure. The subsequent blood flow was measured after deflation. Five measurements were performed by inflating the cuffs to 60 mmHg for 5 s every 10 s. The post-ischemic peak flow (PIPF in ml/min) was defined as the maximum flow within 15 s after ischemia. The post-ischemic flow reserve (PIFR) was defined as the ratio between PIPF and LBF.

Arterial stiffness was assessed by measuring the carotid to femoral PWV, which is considered the gold standard for this purpose and recommended by current guidelines [14, 15]. Patients were supine for at least 10 min prior to the examination. The transit time of the pulse waves between the right carotid artery and the right femoral artery was measured using the Vicorder device (SMT Medical, Würzburg, Germany). The carotid to femoral path length was calculated by subtracting the distance between the carotid recording site to the suprasternal notch from the distance between the femoral recording site via the umbilicus to the suprasternal notch [16]. Distances were determined using a measuring tape. The PWV was calculated by the Vicorder as carotid to femoral path length (m) divided by the transit time of the pulse wave (s). We used the mean PWV out of 3 to 5 measurements.

### Statistical analysis

A priori sample size estimates were calculated with a power analysis based on the results of a previous clinical study, which showed that the PWV was significantly higher in patients after renal transplantation compared to controls [17], assuming that in PKU increased arterial stiffness might be due to a renal dysfunction [5]. With a power of 80% and considering 30% more controls than patients for a better comparison, the calculated sample size was *n* = 21 for PKU patients and *n* = 28 for healthy controls. We used Tukey’s method to exclude outliers [18].

Statistical analyses were performed using IBM SPSS (Windows, Version 24). For non-parametric distribution, data are expressed as median and interquartile range. In these cases, the Mann-Whitney-U-test was used for statistical comparison between the groups and the Spearman-Rho correlation coefficient to explore associations with other variables. For parametric distribution, data are expressed as mean ± standard deviation, and the independent t-test and Pearson correlation coefficient were used for statistical analysis. Multiple linear regression analysis was used to evaluate the influence of measured variables to the outcome variable of interest. A value of *p* < 0.05 was considered significant.

## Results

### Patient characteristics

Of 123 patients regularly attending the outpatient clinic for metabolic disorders at the Charité-Universitätsmedizin Berlin, 29 patients who met the inclusion criteria and gave informed consent were enrolled in the study. 3 patients were excluded after review of their clinical history (patients were smokers) and 3 patients did not complete the study as they had to leave before completion of the investigation. Thus, a total of 23 patients with PKU and 28 healthy controls were included in the final analysis. 19 patients were classified as classical PKU and 4 patients mild PKU. 5 patients were regularly taking anti-hypertensive medication (4 patients monotherapy, one patient combination therapy) and one patient was on statin therapy.

The demographic and clinical characteristics of both groups are shown in Table 1. Complete clinical characteristics were collected only in 21 patients. Two patients refused physical examination but participated in the vascular studies. Age was similar in both groups (PKU patients: 30.8 ± 8.4 y, controls: 30.1 ± 9.1 y) ranging from 18 to 47 years. BMI, resting heart rate and the systolic blood pressure were significantly higher in PKU patients than in healthy controls.

**Table 1:** Clinical characteristics of patients and healthy controls

Characteristics	PKU patients		Control group		*p*-value
	mean ± SD	n	mean ± SD	n	
Male sex - n (%)		13 (57)		11 (39)	
Age - years	30.8 ± 8.4	23	30.1 ± 9.1	28	n.s.
BMI - kg/m^2^	27.6 ± 5.4	21	23.4 ± 6.4	28	**< 0.001**
Heart rate - bpm	72.8 ± 10.5	21	62.1 ± 9.9	27	**0.001**
Mean arterial pressure - mmHg	89.9 ± 7.5	21	84.5 ± 7.1	28	**0.014**
Systolic blood pressure - mmHg	124.6 ± 10.4	21	116.1 ± 10.7	28	**0.012**
Diastolic blood pressure -mmHg	72.5 ± 6.9	21	68.6 ± 7.3	28	**0.023**
Pulse pressure - mmHg	52.1 ± 7.1	21	47.5 ± 10.1	28	n.s.

Renal function was normal in PKU patients (*n* = 22) as assessed by the serum creatinine (0.78 ± 0.13 mg/dl), urea levels (22.5 ± 5.2 mg/dl), eGFR (113.9 ± 13.2 ml/min/1.73 m^2^) and the urine protein-to-creatinine ratio (77.2 ± 17.4 mg/g, *n* = 16; normal: < 140 mg/g).

### Body mass index

PKU patients had a mean BMI of 27.6 ± 5.4 kg/m^2^, which is classified as preobese according to the WHO criteria [19]; 48% of the patients had a weight within the normal range, 19% were preobese and 33% were obese (50% of female and 18% of male patients). BMI was significantly higher compared to healthy controls with a mean BMI in the normal range (mean: 23.4 ± 6.4 kg/m^2^).

The presence of a higher BMI and higher blood pressure raised the question whether these were components of a metabolic syndrome, which is known to be associated with increased arterial stiffness and higher inflammation [20, 21]. We therefore analyzed the fasting plasma glucose in all patients (mean: 84.7 ± 8.1 mg/dl) and reviewed triglyceride levels, HDLc levels and blood pressure, as part of the definition of metabolic syndrome following the National Cholesterol Education Program’s Adult Treatment Panel III report (ATP III) [22]. Only 2 patients fulfilled 2 out of 5 criteria for metabolic syndrome, while 3 positive criteria are necessary for the diagnosis. However, as we did not measure the waist circumference, the diagnosis could not be made. The remaining 21 patients did not meet the criteria of a metabolic syndrome. However, there was an inverse correlation of HDLc levels with the systolic blood pressure (r = − 0.439, *p* = 0.047), indicating an association of these metabolic syndrome components.

### Dietary protein and Phe plasma concentrations

Current Phe plasma concentrations, mean blood Phe plasma concentrations of the previous 5 years (Phe mean 5 y), tyrosine plasma concentrations and protein intake of the PKU patients are shown in Table 2**.** We calculated total protein and current synthetic protein intake (58.1 ± 14.2 g/d, 72.8 ± 12.4 g/d) and the mean of the synthetic and total protein intake of the last 5 years per kilogram body weight (0.75 ± 0.22 g/kg/d, 0.94 ± 0.21 g/kg/d). There were no significant associations between dietary intake parameters (Table 2) and vascular measurements, inflammatory or oxidative stress markers and serum lipoproteins.

**Table 2:** Plasma amino acid levels and dietary intake in PKU patients

	mean ± SD	n
Phenylalanine – μmol/l	1172 ± 380	21
Tyrosine – μmol/l	85.6 ± 38.5	21
Phe mean 5y – μmol/l	1027 ± 263	22
Current total protein intake - g/d	72.8 ± 12.4	19
Current synthetic protein intake - g/d	58.1 ± 14.2	19
Current total protein intake - g/kg/d	0.92 ± 0.20	18
Current synthetic protein intake - g/kg/d	0.73 ± 0.21	18
Total protein intake mean of 5y - g/kg/d	0.94 ± 0.21	18
Synthetic protein intake mean of 5y - g/kg/d	0.75 ± 0.22	18

Phe plasma levels significantly and positively correlated with the BMI (r = 0.689, *p* = 0.001) and the concentration of non-HDLc (r = 0.547, *p* = 0.010), triglyceride (r = 0.452, *p* = 0.040), and the LDLc/HDLc-Ratio (r = 0.614, *p* = 0.003), respectively and negatively with the HDL2c concentration (r = − 0.484, *p* = 0.026) and the HDL2c/3c-ratio (r = − 0.497, *p* = 0.022).

Patients with poorly controlled PKU,defined as current Phe concentration higher than 1200 μmol/l, according to the German PKU recommendations [23], had higher serum lipid levels and a higher BMI, but there was no significant difference in other blood parameters and the vascular measurements (Table 3)**.** Phe plasma concentrations did not correlate with biomarkers of oxidative stress or inflammation or with vascular measurements.

**Table 3:** Significantly different clinical and laboratory parameters of well * (Phe < 1200 μmol/l; *n* = 13) and poorly (Phe > 1200 μmol/l; *n* = 8) controlled patients

	Well controlled patients	Poorly controlled patients	
	mean ± SD	mean ± SD	*p*-value
Phe μmol/l	920.2 ± 163.4	1580.0 ± 254.3	**< 0.001**
Phe mean 5y - μmol/l	829.3 ± 139.2	1247.0 ± 163.4	**< 0.001**
Total protein intake mean of 5y - g/kg/d	1.0 ± 0.2	0.7 ± 0.0	**0.022**
Age - years	28.3 ± 8.7	35.3 ± 6.9	**0.050**
BMI - kg/m^2^	24.0 ± 2.3	32.1 ± 5.3	**0.001**
LDLc/HDLc	1.9 ± 0.5	2.3 ± 0.5	**0.025**
Triglycerides - mmol/l	1.0 ± 0.4	1.9 ± 1.1	**0.005**
Non-HDLc - mmol/l	2.7 ± 0.4	3.2 ± 0.3	**0.017**
HDL2c - mmol/l	0.7 ± 0.3	0.5 ± 0.2	n.s.
HDL2c/HDL3c	0.9 ± 0.3	0.6 ± 0.3	n.s.

*according to the German PKU recommendations [23]

### Lipoprotein profile

Table 4 shows the serum levels of total lipids, lipoprotein lipids and apolipoproteins. PKU patients had significantly lower HDLc, lower HDL2c and higher non-HDLc levels, altogether indicating an atherogenic pattern [24, 25]. The non-HDLc levels (r = 0.486, *p* = 0.026), but no other blood lipid levels were significantly associated with the BMI.

**Table 4:** Lipoproteins, markers of inflammation and oxidative stress in patients and healthy controls

	PKU patients		Control group		*p*-value
	mean ± SD or median [IQR]	n	mean ± SD or median [IQR]	n	
Lipids and Lipoproteins (mmol/l)					
Total Cholesterol	4.3 ± 0.6	23	3.8 ± 0.6	28	**0.003**
LDLc	2.8 ± 0.7	23	2.6 ± 0.8	28	n.s.
HDLc	1.4 ± 0.3	23	1.6 ± 0.3	28	**0.032**
LDLc/HDLc	2.1 ± 0.5	23	1.7 ± 0.6	28	**0.018**
ApoA1 (mg/ml)	2.1 ± 0.7	23	1.8 ± 0.3	16	n.s.
HDL2c	0.6 ± 0.3	23	0.8 ± 0.2	28	**0.009**
HDL3c	0.8 ± 0.1	23	0.8 ± 0.1	28	n.s.
HDL2c/HDL3c	0.8 ± 0.3	23	1.1 ± 0.3	28	**0.005**
Non-HDLc	2.9 ± 0.4	23	2.2 ± 0.6	28	**< 0.001**
Triglycerides	1.3 ± 0.8	23	1.1 ± 0.6	28	n.s.
Phospholipids	2.7 ± 0.6	23	2.6 ± 0.4	28	n.s.
Markers of Inflammation (mg/l)					
SAA	6.7 [2.8–14.4]	22	2.9 [1.5–5.2]	27	**0.010**
CRP	18.4 [7.3–83.3]	22	6.3 [2.0–42.1]	27	**0.010**
Markers of Oxidative Stress					
MDA (μg/ml)	0.33 [0.23–0.59]	23	0.23 [0.12–0.38]	27	**0.027**
MPO (μg/ml)	0.68 [0.29–0.97]	19	0.89 [0.34–1.3]	26	n.s.
3-NT (nmol/l)	1.8 [0.34–2.7]	22	1.7 [0.92–2.4]	28	n.s.

The HDL2c levels were negatively correlated with the systolic blood pressure (r = − 0.470, *p* = 0.031, *n* = 21), Phe plasma concentrations (r = − 0.484, p = 0.026) and MDA (r = − 0.580, *p* = 0.004) and positively with SAA (r = 0.517, *p* = 0.014, *n* = 22) in PKU patients. This suggests associations of the altered cholesterol content within the HDL fractions with dietary adherence, inflammation and oxidative stress.

### Inflammation and oxidative stress

**Fig. 1:**
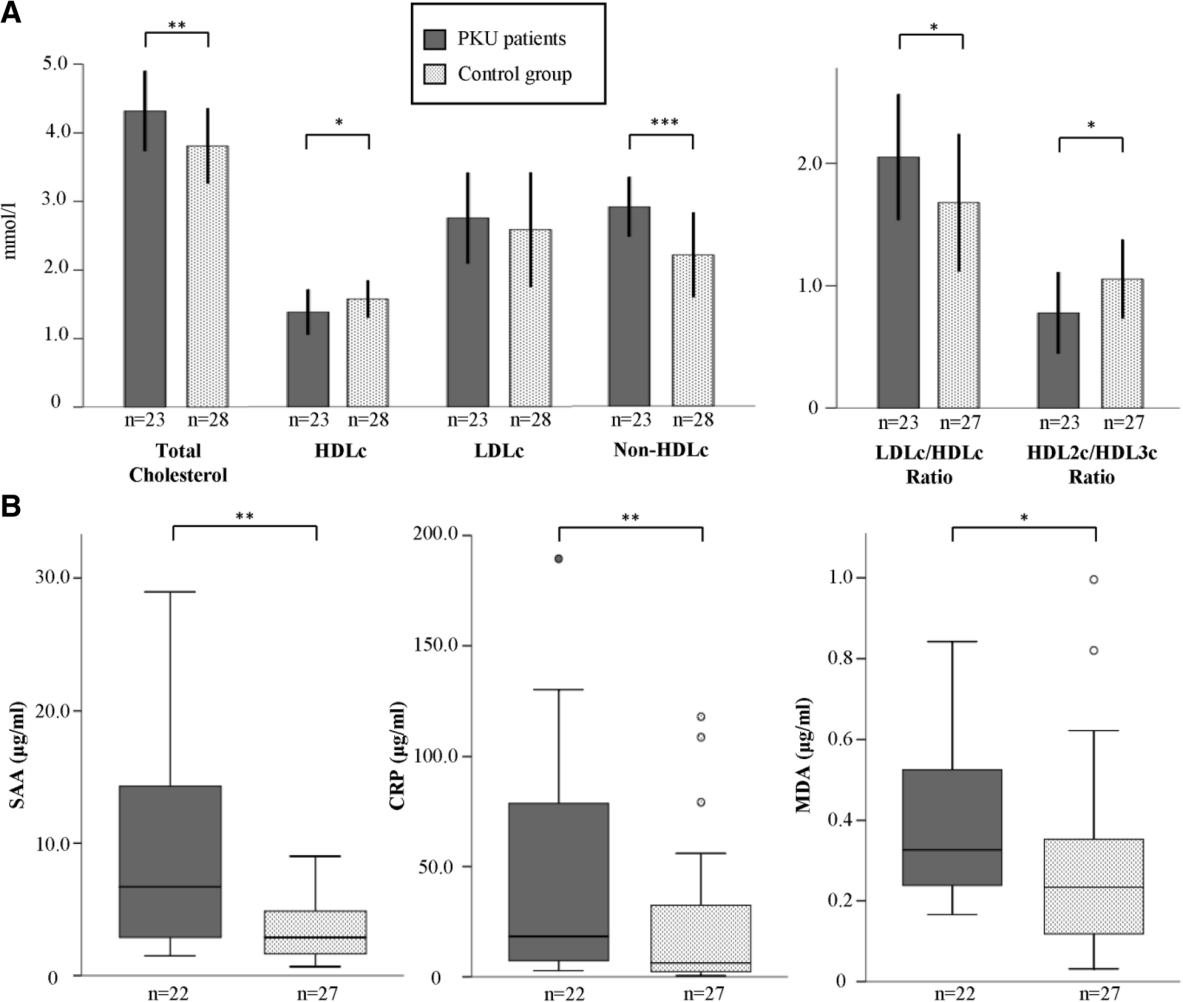
**a** Serum lipoprotein lipids in patients with phenylketonuria (PKU) and healthy controls. Bar charts showing the mean and SD. *p < 0.05, **p ≤ 0.01, ***p ≤ 0.001 (Unpaired T-Test). **b** Box-plot presentation of SAA, CRP and MDA serum levels in patients with phenylketonuria (PKU) and healthy controls. *p < 0.05, **p ≤ 0.01 (Mann-Whitney-U-Test)

As further atherogenic risk factors, we investigated serum biomarkers for inflammation, SAA and CRP, and oxidative stress, MDA, MPO, 3-NT (Table 4, Fig. 1). Both SAA and CRP serum concentrations were elevated 2–3-fold in PKU patients compared to controls and were significantly correlated with the BMI (CRP: r = 0.426, *p* = 0.003; SAA: r = 0.322, *p* = 0.027), suggesting an influence of the increased weight on the patient’s inflammatory status.

Similarly, MDA levels were significantly higher in PKU patients, but there were no significant differences of MPO and 3-NT levels between the groups.

We further analyzed serum selenium concentrations as part of the antioxidatively acting glutathione peroxidase, which were found to be negatively correlated with elevated MDA levels in other studies [26, 27]. However, selenium levels were in a normal range (0.84 ± 0.18 μmol/l) and there was no correlation with MDA levels.

Taken together, these data indicate the presence of inflammation and oxidative stress in the PKU patients.

### Vascular studies

**Fig. 2:**
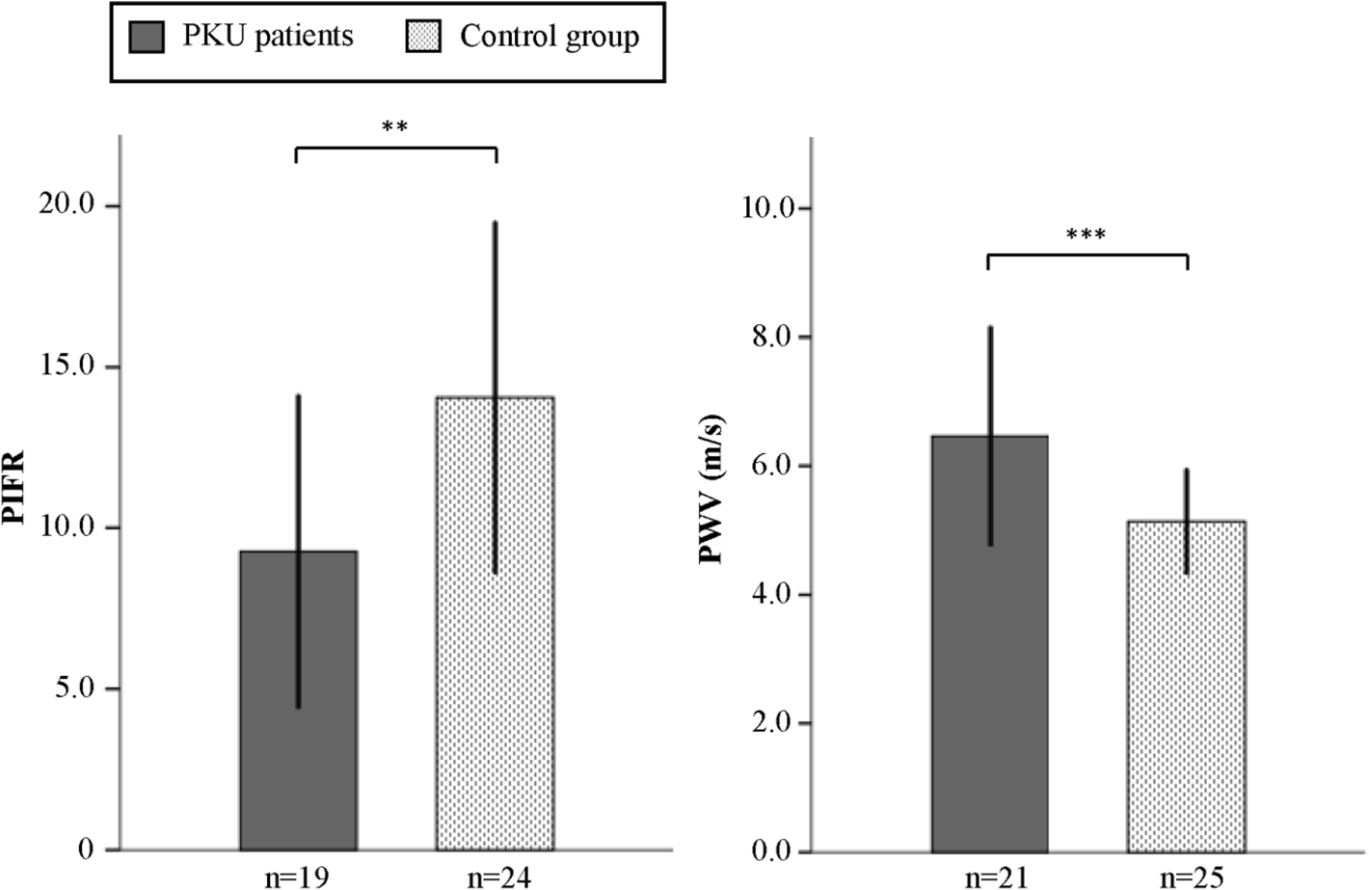
Post-ischemic flow reserve (PIFR) and pulse wave velocity (PWV) in patients with phenylketonuria (PKU) and healthy controls. Bar charts showing the mean and SD. **p ≤ 0.01, ***p ≤ 0.001 (Unpaired t-test)

Endothelial dysfunction was evident in the PKU patients, who had a significantly lower PIFR than the healthy controls (9.3 ± 4.9 versus 14.1 ± 5.5, *p* = 0.005; Fig. 2). Endothelial function was also inversely associated with the BMI (r = − 0.50, *p* = 0.028), but not with systolic or diastolic blood pressure in the PKU patients. A multiple regression analysis was performed to test whether the observed differences in endothelial function between the groups were independently affected by the BMI. Only the BMI (but not the diagnosis of PKU) was a significant predictor of PIFR (β = − 0.50 *p* = 0.002, R^2^ = 0.32), suggesting that endothelial dysfunction in the PKU patients was mediated by their increased BMI. In some patients and controls, no reliable vascular measurements could be performed.

Arterial stiffness was significantly increased in PKU patients compared to controls, as indicated by the PWV (6.4 ± 1.7 m/s versus 5.1 ± 0.8 m/s, p = 0,001; Fig. 2). As expected, PWV correlated with age (r = 0.756, *p* < 0.001), but there was no correlation with the systolic blood pressure or the BMI. A multiple regression analysis (R^2^ = 0.49) showed that both, age (β = 0.55, p < 0.001), and the diagnosis of PKU (β = − 0.48, p < 0.001) were independent predictors of the PWV.

Altogether, these vascular studies showed endothelial dysfunction and an increased arterial stiffness in patients with PKU, representing subclinical disturbances in both vascular function and structure.

## Discussion

This study confirms the presence of classical risk factors for cardiovascular disease in adult patients with PKU, including an increased blood pressure, obesity and an atherogenic lipoprotein profile. In addition, we found an increase in resting heart rate, elevated biochemical indicators of inflammation and oxidative stress, marked endothelial dysfunction and increased vascular stiffness. Risk factors and biochemical markers (LDLc/HDLc, triglycerides, non-HDLc, HDL2c) were in part modified by dietary adherence as indicated by the current plasma Phe concentrations. These data further define the cardiovascular phenotype of adult PKU patients and suggest an important pathophysiological role for dietary adherence.

The patients in this study had higher **Phe** plasma concentrations than recommended in the European guidelines [28] and also compared to other studies [4, 6]. Indeed, poor adherence to the strict PKU diet and consequently high Phe concentrations are a well-known problem in patients with PKU [28]. In a recent multicenter survey, most patients achieved Phe blood concentrations < 1200 μmol/l, although there was limited consensus regarding target Phe levels for adult PKU patients [29]. The majority of surveyed adult patients in another multicenter study admitted poor compliance with the Phe-restricted diet, which seems to reflect worsening of metabolic control with age reported in several studies [30]. Thus, the rather high Phe plasma concentrations in our study patients most likely indicate poor compliance with dietary advice. However, Phe plasma concentrations in our study were not associated with cardiovascular measurements, including systolic or diastolic blood pressure, resting heart rate, endothelial function and PWV.

Our patient’s characteristics were comparable with other studies of adult PKU patients, not only including increased Phe plasma concentrations but also concerning dietary data and a high **BMI** [4, 29, 31]. In our patients, the total protein intake, intake of synthetic protein and the BMI (i.e. 27.6 kg/m^2^) were almost identical to adult PKU patients included in a recent study of body composition of PKU patients in the US [31] . In another large study of 236 British adult PKU patients, a similar mean BMI (26 kg/m^2^) was reported, which increased with age and was significantly correlated with the Phe level [4]. In our study, the BMI was associated with Phe plasma concentrations, confirming previous studies in adult PKU patients, while such association was not found in children with PKU [6]. Furthermore, the BMI was associated with inflammatory markers (CRP, SAA) and non-HDLc levels; it correlated with endothelial dysfunction, but not with other cardiovascular measurements (blood pressure, resting heart rate and PWV). This is in contrast to the well-known association of BMI with systolic and diastolic blood pressure in the general population [32].

Our study confirms several previous reports of a high prevalence of **obesity** in the adult PKU population [4, 11, 33], with 52% of the patients preobese or obese. In contrast, Rocha et al. found a much lower mean BMI (20.1 kg/m^2^) and no evidence of obesity in their study of body composition of 89 patients [34]; however, the majority of their patients was much younger (mean age 14.4 years) and had milder forms of PKU.

We found significantly higher systolic and diastolic blood pressure as well as a significantly increased resting heart rate. While some studies in children and young adults with PKU found normal or even lower **blood pressure** compared to controls [11, 35], a significantly higher blood pressure was found in obese PKU children [7] and in a study of renal function in adolescents and young adults [5]. To our knowledge an increased resting heart rate, a predictor of cardiovascular mortality in the general population [36], has not been described before in PKU patients.

The **lipoprotein profile** in PKU patients was significantly different from the control group. Patients had higher levels of total cholesterol and non-HDLc and a higher LDLc/HDLc ratio, while HDLc levels were lower. This pattern is widely used for the definition of dyslipidemia and generally indicates a higher risk for cardiovascular disease [37]. Importantly, the (elevated) non-HDLc lipid fraction, but not the (decreased) HDLc, was positively associated with the BMI, which is at variance with the most common finding in the general population, which typically shows strong correlations between obesity and (elevated) triglyceride levels and (decreased) HDLc [38]. Instead, the low HDLc levels were inversely associated with inflammation parameters and with Phe plasma levels in our patients; this seems to indicate complex influences on the lipoprotein profile in adult PKU patients.

Previous studies of **total cholesterol** levels in PKU patients have shown inconsistent results. While in some studies lower cholesterol levels were measured [9, 39], we and others found higher cholesterol levels in PKU patients [40]. Lower HDLc levels have been described before in children and adults with PKU [6, 41].

The **PKU diet** in adults is similar to a vegetarian diet, avoiding protein mainly of animal origin, and supplemented by a synthetic Phe-free amino acid mixture [39]. Patients have a low natural protein intake, a low fat intake and a high carbohydrate intake [39]. A vegetarian diet is beneficial for cardiovascular health [42] and associated with decreased levels of total cholesterol, LDLc and HDLc [43]. Therefore, the low HDLc and HDL2c levels in our patients could be attributed at least in part to the PKU diet and in this context, the low HDLc levels and the elevated LDLc/HDLc ratio may not be interpreted as a classical cardiovascular risk factor [39]. However, the negative associations of HDLc and HDL2c with Phe plasma levels and MDA levels indicate that HDLc lipid parameters were independently influenced not only by dietary non-adherence (higher Phe plasma concentrations) but also by higher oxidative stress.

**HDL** is a lipoprotein which has been shown to be inversely correlated to the risk of cardiovascular disease [44]. Its antiatherogenic capacity derives mainly from its function to promote cholesterol efflux from cells (i.e. lipid laden macrophages) [45].

HDL ameliorates endothelial functions and has anti-inflammatory properties. It can be classified into subtypes differing in density [46]. HDL2c has been suggested a more adequate risk indicator for cardiovascular disease in the general population than HDLc and HDL3c [24], although other studies could not confirm an association of CVD with HDL subclasses [47]. **HDL subtypes** have not been studied previously in PKU patients. We found that the decrease in HDLc was due to low cholesterol levels in the HDL2, but not the HDL3 fraction. Studies analyzing the HDL subtypes in vegetarian populations also found a decrease in the HDL2 fraction, probably due to lower cholesterol intake and a higher polyunsaturated-to-saturated fat ratio [48]. Thus, low HDLc and HDL2c levels in our patients were negatively correlated with Phe plasma levels, suggesting that a low adherence to the PKU diet with high Phe plasma concentrations lowered cholesterol content of the HDL2 fraction. We found an inverse correlation between MDA and HDLc as well as HDL2c in patients with PKU. MDA has been supposed to alter the HDL mediated cholesterol efflux by modifying Apo A1 and can thereby lead to dysfunctional HDL [49].

Oxidative stress as well as high SAA can alter the antiatherogenic properties of HDL [45, 50]. Importantly, HDL may acquire pro-inflammatory functions by enrichment with SAA [50]. We observed a strong positive correlation between HDLc and SAA suggesting an enrichment of SAA in HDL, which could possibly indicate an altered function of HDL in PKU patients.

Beside the traditional cardiovascular risk markers, we further analyzed **oxidative stress** and inflammation in PKU patients as possible mediators of endothelial dysfunction and vascular stiffness. Biomarkers included MDA, a marker of lipid peroxidation, the peroxidase MPO, and 3-NT, reflecting tyrosine oxidation by reactive nitrogen species. We found that MDA-levels, but not MPO or 3-NT, were increased in patients with PKU. This supports the results of Ercal et al. [51], who found increased MDA levels in a PKU animal model. Furthermore Wilke et al. [27] found higher MDA levels in children with PKU, which were reversible after selenium substitution. Selenium, as part of the antioxidatively acting glutathione peroxidase, was found to be reduced in PKU patients [26]. However, our patients had normal selenium levels, indicating the presence of increased oxidative stress independent of the serum selenium status. These data support previous studies demonstrating oxidative stress in patients with PKU [8, 41]. The underlying causes of increased oxidative stress in PKU are still a matter of debate, but increased Phe plasma concentrations have been found associated with several markers of oxidative stress in other studies [52, 53]. Possible mechanisms proposed for oxidative stress in patients with PKU include the high blood concentrations of phenylalanine, which may directly induce oxidative damage but also a decreased antioxidative defense as result of the strict diet leading to a lack of macro- and micronutrients with antioxidative functions [8, 54].

While oxidative stress is well documented in PKU patients, suggesting a high pro-inflammatory potential, markers of **inflammation** have been rarely studied thus far. Deon et al., could demonstrate increased serum levels of IL-1b, IL-6 and IL-10 in a study of 7 well-controlled adolescent patients with PKU compared to controls [10]. In our study, CRP and SAA in serum were elevated and associated with the BMI, but not with Phe levels. It is very well established that SAA plays a relevant role for HDL-functionality, endothelial dysfunction and progression of atherosclerosis [55]. In addition, there was a significant correlation between a higher total protein intake and lower CRP, suggesting a suppressive effect of dietary adherence on inflammation. Altogether these data indicate an obesity-induced inflammatory status in the patients included in our study.

We further analyzed the vascular status and found alterations in its function and structure.

To our knowledge, **endothelial function** has not been studied previously in patients with PKU. We here show a significant reduction of post-ischemic blood flow in these patients as measured by venous occlusion plethysmography, a long established and validated method [56]. The post-ischemic flow reserve (PIFR) was reduced by 34%, indicating striking endothelial dysfunction, which was associated with the BMI, as shown in the multivariate analysis. Obesity is a well-known cause of endothelial dysfunction, mainly mediated by inflammation and oxidative stress [57].

A decline of the elastic properties of the aorta is a result of vascular ageing and leads to an acceleration of the pulse wave. Thus, a high PWV can be interpreted as a sign of relevant arterial damage. The development of vascular stiffness in the general population is strongly promoted by hypertension, inflammation and oxidative stress [58], but it is unknown whether PKU itself or dietary treatment of PKU may affect arterial properties by specific metabolic mechanisms. In a small study of vegetarian men, carotid intima-media thickness and distensibility and vascular stiffness measured by PWV were reduced by the vegetarian diet [59]. In our study, patients with PKU had a significant increase in vascular stiffness, which was independent of blood pressure or BMI but associated with the serum CRP. Hermida-Ameijeiras et al. found a similar increase in PWV (classical PKU only) in a study of 41 PKU patients (age 6–50 years; mean age 23; 61% overweight, 39% obese) who had no significant changes in blood pressure, heart rate and blood lipid levels [11]. In their study, PWV was associated with several other variables including age, BMI, central diastolic blood pressure, and median Phe plasma concentrations; however, significant predictors of PWV in a multivariate analysis were age and central blood pressure. In another study Htun et al. found an increase in carotid intima media thickness and local vascular stiffness [60], which is in accordance with our results.

Taken together, these data indicate endothelial dysfunction and premature vascular ageing in PKU patients. Increased oxidative stress and a decrease in antioxidative activity has been demonstrated in PKU patients [53], which along with the presence of inflammation might promote vascular damage. Although we could not find a significant association of PWV with oxidative stress, it should be noted that our observations were not targeted to comprehensively analyze oxidative stress or inflammation but were limited to few selected makers. Further limitations include the small number of patients and the lack of additional studies of validated surrogate markers for cardiovascular disease, such as echocardiography or measurement of intima-media thickness, which could not be performed in this study for logistical reasons.

In summary, this study provides evidence for an increased cardiovascular risk in PKU patients. An accumulation of traditional cardiovascular risk factors, high inflammatory and oxidative stress markers, endothelial dysfunction and vascular stiffness characterize the cardiovascular phenotype of adult PKU patients. These findings indicate the need for cardiovascular monitoring and early preventive measures against cardiovascular disease in patients with PKU.

Recent guidelines by the American Heart Association provide detailed recommendations for risk calculation and treatment of atherosclerotic cardiovascular disease (ASCVD) for the general population [61]. For adult PKU patients, we therefore suggest to consider calculating the individual risk by ASCVD-calculator and to follow the primary prevention concept of the 2018 AHA recommendation. A statin therapy should be considered in patients with PKU with an estimated ASCVD risk higher than 5%.

## Data Availability

The datasets used and/or analysed during the current study are available from the corresponding author on reasonable request.

## Abbreviations

3-NT: 3-nitrotyrosine
apoA1: apolipoprotein A1
BMI: Body mass index
CRP: C-reactive protein
eGFR: estimated glomerular filtration rate
HDL: High density lipoprotein
HDLc: HDL cholesterol
LBF: Low baseline blood flow
LDL: Low density lipoprotein
MDA: Malondialdehyde
MPO: Myeloperoxidase
n. s.: non-significant
Phe mean 5y: Mean blood Phe plasma concentrations of the previous 5 years
Phe: Phenylalanine
PIFR: Post-ischemic flow reserve
PIPF: Post-ischemic peak flow
PKU: Phenylketonuria
PWV: Pulse wave velocity
SAA: Serum amyloid A
VOP: Venous occlusion plethysmography
y: years
